# Development and Validation of a Machine Learning Prognostic Model for Hepatocellular Carcinoma Recurrence After Surgical Resection

**DOI:** 10.3389/fonc.2020.593741

**Published:** 2021-02-01

**Authors:** Yao Huang, Hengkai Chen, Yongyi Zeng, Zhiqiang Liu, Handong Ma, Jingfeng Liu

**Affiliations:** ^1^ Liver Disease Center, The First Affiliated Hospital of Fujian Medical University, Fuzhou, China; ^2^ The United Innovation of Mengchao Hepatobiliary Technology Key Laboratory of Fujian Province, Mengchao Hepatobiliary Hospital of Fujian Medical University, Fuzhou, China; ^3^ The Liver Center of Fujian Province, Fujian Medical University, Fuzhou, China; ^4^ Department of Computer Science, Shanghai Jiao Tong University, Shanghai, China

**Keywords:** hepatocellular carcinoma, recurrence, machine learning, modeling, prognosis

## Abstract

Surgical resection remains primary curative treatment for patients with hepatocellular carcinoma (HCC) while over 50% of patients experience recurrence, which calls for individualized recurrence prediction and early surveillance. This study aimed to develop a machine learning prognostic model to identify high-risk patients after surgical resection and to review importance of variables in different time intervals. The patients in this study were from two centers including Eastern Hepatobiliary Surgery Hospital (EHSH) and Mengchao Hepatobiliary Hospital (MHH). The best-performed model was determined, validated, and applied to each time interval (0–1 year, 1–2 years, 2–3 years, and 3–5 years). Importance scores were used to illustrate feature importance in different time intervals. In addition, a risk heat map was constructed which visually depicted the risk of recurrence in different years. A total of 7,919 patients from two centers were included, of which 3,359 and 230 patients experienced recurrence, metastasis or died during the follow-up time in the EHSH and MHH datasets, respectively. The XGBoost model achieved the best discrimination with a c-index of 0.713 in internal validation cohort. Kaplan-Meier curves succeed to stratify external validation cohort into different risk groups (p < 0.05 in all comparisons). Tumor characteristics contribute more to HCC relapse in 0 to 1 year while HBV infection and smoking affect patients’ outcome largely in 3 to 5 years. Based on machine learning prediction model, the peak of recurrence can be predicted for individual HCC patients. Therefore, clinicians can apply it to personalize the management of postoperative survival.

## Introduction

Hepatocellular carcinoma (HCC) is the most common primary liver cancer and ranks as the fourth leading cause of cancer-related mortality (8.2%) worldwide (1). Surgical resection remains the primary curative treatment for patients with adequate liver function (2). However, 50% to 70% of patients who undergo complete tumor resection still suffer from frequent recurrence and disease progression, ultimately leading to unfavorable prognoses (3). Therefore, the identification of patients at high risk of recurrence after surgical resection is essential for clinicians to provide appropriate surveillance and therapy.

During the past decade, researchers have primarily focused on prognosis-predictive models based on biological, demographic, and clinical factors. The most acknowledged system of the American Joint Committee on Cancer (AJCC) tumor-node-metastasis (TNM) is commonly used to determine the staging of liver cancer. However, its prognostic value in predicting tumor recurrence is widely debated (4). Recent models, including the Singapore Liver Cancer Recurrence (SLICER) score, Surgery-Specific Cancer of the Liver Italian Program (SS-CLIP), and the Korean model, were designed to detect tumor recurrence in specific groups of patients. Due to the inaccuracy and diversity of these models, they have not been widely implemented (5–7). In addition, the Early Recurrence After Surgery for Liver tumor (ERASL) model, which is based on Cox regression analysis, has been established to predict early tumor recurrence after liver resection. Despite its better discriminatory performances than other tools, the limited clinical parameters and the prediction for 2-year recurrence restrict its application in the full HCC survivorship management (8).

Machine learning, a field of computer science in which machines mimic, recognize, and learn cognitive functions of the human mind to make empirical predictions, is gaining more and more attention in recent years (9). For cancer, machine learning demonstrates the advantages of image recognition and feature selection compared to traditional methods (10, 11). Recently, automated machine learning algorithms have been developed to detect metastasis in sentinel lymph nodes of women with breast cancer, and showed better diagnostic performance than pathologists (12). In patients with bladder cancer, a novel predictive model based on machine learning algorithms was also created. In the model, disease recurrence after cystectomy was predicted with more than 70% sensitivity and specificity (13). However, few studies have applied a machine learning framework to identify HCC patients with the potential risk of recurrence after curative treatment.

Briefly, we aimed to utilize machine learning algorithms to develop a risk prediction model to predict HCC recurrence among patients who underwent surgical resection. We also explored feature importance in this process, verifying the important prognostic factors for tumor relapse. In addition, a risk heat map covering five years that visually depicts the risk of recurrence was constructed. In this way, we hope to improve the performance of HCC recurrence predictive models using big data and to provide evidential support for individualized management.

## Materials and Methods

This analysis was reported according to the TRIPOD (Transparent Reporting of a Multivariable Prediction Model for Individual Prognosis or Diagnosis) guidelines (14).

### Patients

The database was retrospectively derived from patients with HCC who underwent hepatic resection at Eastern Hepatobiliary Surgery Hospital, Second Military Medical University (EHSH) (n = 7,411, from May 2008 to Sept. 2018) or Mengchao Hepatobiliary Hospital, Fujian Medical University (MHH) (n = 508, from Nov. 2014 to Nov. 2018). The patients in this study met the inclusion criteria as follows: (1) pathological confirmation of HCC, (2) Child-Pugh A/B before surgery, (3) R0 surgical resection of tumor with curative intent. However, patients who (1) died within 30 days after surgery or lost to follow-up, (2) received preoperative neoadjuvant treatment (3) diagnosed with extrahepatic cancers, HCC relapse, or metastasis (4) younger than 18 years old were excluded from this study. Inclusion and exclusion of patients and following analysis can be found in **Supplementary Figure 1**.

Different models were constructed on the EHSH dataset, which was randomly divided into derivation and internal validation cohorts at a ratio of 8:2. The models were validated externally using the dataset from MHH. The study was approved by the Ethics Committee of the two centers, and the requirement of written informed consent was waived. All procedures were performed in accordance with the Declaration of Helsinki.

### Clinical Variables

The demographics, laboratory tests, and HCC etiologies were collected from the database. The laboratory tests included various parameters of blood examination, liver and coagulation function, and hepatitis virus markers. Tumor characteristics included, but were not limited to, the number of tumors, the diameter of the largest nodule, differentiation, capsule, cirrhosis in non-cancerous tissues, and vascular invasion. Macrovascular invasion was defined as tumor invasion of large vessels, which can be detected by Computed Tomography/Magnetic Resonance Imaging (CT/MRI) (8). Microvascular invasion refers to the histologically microscopic presence of cancer cell clusters in the blood vessels lined with endothelial cells (15). Thirty-five variables were selected by health professionals based on literature review and clinical expertise.

### Follow-up and Outcome

During the follow-up, serum alpha-fetoprotein (AFP) levels were measured, as well as ultrasonography, CT, or MRI of the chest and abdomen once every two months for six months, and then once every three months for the next 1.5 years. For patients who were free of cancer recurrence two years after surgery, a 6-month interval surveillance was carried out. The outcome of this study, recurrence-free survival (RFS), was defined as the time from surgery to the detection of recurrence, metastasis, or death.

### General Statistical Principle

After preliminary data cleaning, multiple imputation was performed in R (v3.6.2) based on the Multivariate Imputation by Chained Equations (MICE, v3.8.0). Continuous variables, which were tested for normality by Anderson-Darling tests, were abnormally distributed. Therefore, the variables were summarized by median (IQR), and Wilcoxon rank-sum tests were used for between-groups comparisons. Categorical variables were expressed as frequency (%), and Chi-squared tests or Fisher’s exact tests were applied, as appropriate. All statistical analyses above were two-sided, while p < 0.05 was considered statistically significant, and conducted in Python (v3.7) with Scipy (v1.4.0) package.

### Model Development

#### Cox Proportional Hazards Model (CPH)

The clinicopathologic parameters of HCC recurrence were fitted by the Cox regression using the Survival package (v3.1) in R-language. Univariable Cox regression was firstly conducted to identify potential predictors (p < 0.1). Variables identified in univariable cox model were then applied in multivariable cox regression with stepwise selection method.

#### Machine Learning Models

Three machine learning models, including Deep Learning-based Survival Model (DeepSurv), Extreme Gradient Boosting (XGBoost), and Random survival forest (RSF) were applied to perform the task of predicting HCC recurrence using all 35 variables preselected. DeepSurv is a multi-layer feed-forward neural network that predicts the effects of diverse variables on their hazard rate parameterized by the weights of the network (16). Based on its algorithm principle, we redeveloped DeepSurv in Python under Pytorch deep learning framework (version 1.3.1, CPU version) and optimized the hyper-parameter search. XGBoost is an improved supervised learning algorithm based on the Gradient Boosting Decision Tree algorithm, which can deal with survival problems by setting partial likelihood functions of the optimization object and log-rank tests as node split criteria (17). Our XGBoost model was implemented in Python using the XGBoost (v.0.9) package. RSF is another machine learning approach for survival analysis that eliminates the proportional hazard assumption and can fit a more general spectrum of survival problems, which conducted in R (randomForestSRC v2.9.3) (18).

### Model Discrimination and Calibration

The discrimination performance among the four models in both derivation and validation sets were measured by Harrell’s c-index. Comparison of c-index among different models in each cohort was conducted afterwards (19).

As suggested by previous study Kaplan-Meier survival curves for various risk groups were used as informal evidence of discriminative ability (20). Kaplan-Meier curve for the external validation cohort after calibration allows a visual comparison of discrimination among different risk groups at the cut-off of 50th and 84th centiles.

Calibration plots of XGBoost were applied to the derivation and validation sets to determine whether each patient’s predicted risk was consistent with the actual outcome. We followed the practice of Chan et al. to draw the calibration plots (8) at 1, 2, 3, and 5 years.

### Models in Different Time Intervals and Predictive Heat Map

Inspired by lifetable methodology, we applied XGBoost to different time intervals, including 0 to 1 year, 1 to 2 years, 2 to 3 years, and 3 to 5 years, with the same software. Importance scores were exported, and the Harrell’s c-index of each interval were reported at the same time. Furthermore, fifty patients from the external validation cohort were randomly selected to create a heat map for visually illustrating the risk of recurrence within five years after surgery, with aim of providing guidance and support in clinical practice.

## Results

### Clinicopathologic Features and Outcome

A total of 7,919 patients who underwent surgical resection from two centers were included in the study. 80% of EHSH cohort was assigned as the derivation set (n = 5,928) and the rest was designated as internal validation set (n = 1,483). By the time of data analysis, 3,359 and 230 patients experienced recurrence, metastasis or died during the follow-up time in the EHSH dataset and MHH datasets, respectively. Median follow-up period for two datasets were 3.51 (IQR: 0.41–8.32) and 2.04 (IQR: 0.23–3.88) years. Detailed outcome descriptions are provided in **Supplementary Table 1**.

Thirty-five predictors were included in the final analysis. Preoperative clinical and postoperative pathologic characteristics of the three cohorts are shown in **Table 1**.

**Table 1:** Baseline characteristics of patient.

	EHSH derivation (n = 5,928)	EHSH validation (n = 1,483)	MHH validation (n = 508)	p-value*
**Gender, male, n (%)**	5096 (86.0%)	1305 (88.0%)	437 (86.0%)	0.825
**Age (years), median (IQR)**	52.0 (44.0–60.0)	51.0 (44.0–59.0)	56.0 (48.0–63.2)	<0.001
**Smoking, n (%)**	2278 (38.4%)	587 (39.6%)	121 (23.8%)	<0.001
**Alcohol consumption, n (%)**	1215 (20.5%)	293 (19.8%)	53 (10.4%)	<0.001
**FLD, n (%)**	209 (3.5%)	72 (4.9%)	96 (18.9%)	<0.001
**Ascites, n (%)**	149 (2.5%)	45 (3.0%)	43 (8.5%)	<0.001
**Cirrhosis, n (%)**	5126 (86.5%)	1261 (85.0%)	497 (97.8%)	<0.001
**ALBI grade, n (%)**				<0.001
**1**	4546 (76.7%)	1122 (75.7%)	215 (42.3%)	
**2**	1379 (23.3%)	361 (24.3%)	293 (57.7%)	
**3**	3 (0.1%)	0 (0.0%)	0 (0.0%)	
**Child-Pugh score**				0.003
**A**	5843 (98.6%)	1468 (99.0%)	493 (97.0%)	
**B**	85 (1.4%)	15 (1.0%)	15 (3.0%)	
**HBV history, n (%)**	5307 (89.5%)	1334 (90.0%)	206 (40.6%)	<0.001
**HBV-DNA load (IU/ml), median (IQR)**	1000.0 (1000.0–58000.0)	1000.0 (1000.0–56100.0)	1280.0 (500.0–48400.0)	<0.001
**HBsAg, n (%)**	5028 (84.8%)	1258 (84.8%)	206 (40.6%)	<0.001
**HBsAb, n (%)**	809 (13.6%)	207 (14.0%)	55 (10.8%)	0.066
**HbcAb, n (%)**	5789 (97.7%)	1449 (97.7%)	493 (97.0%)	0.376
**HBeAg, n (%)**	1474 (24.9%)	365 (24.6%)	114 (22.4%)	0.230
**HBeAb, n (%)**	4222 (71.2%)	1075 (72.5%)	358 (70.5%)	0.629
**AFP (ng/ml), median (IQR)**	95.7 (6.5–1210.0)	76.5 (6.7–1210.0)	61.8 (6.2–905.9)	0.148
**GGT (IU/L), median (IQR)**	66.0 (37.0–120.0)	64.0 (36.0–113.4)	53.0 (30.8–102.0)	<0.001
**TBIL (μmol/L), median (IQR)**	13.4 (10.3–17.2)	13.4 (10.4–17.5)	15.7 (11.1–21.5)	<0.001
**Albumin (g/L), median (IQR)**	41.8 (39.4–44.2)	42.0 (39.3–44.2)	39.0 (36.0–42.0)	<0.001
**HBG (g/L), median (IQR)**	143.0 (132.0–152.0)	144.0 (134.0–152.0)	144.0 (132.0–152.0)	0.586
**Prealbumin (mg/L), median (IQR)**	218.0 (177.0–264.0)	225.0 (178.0–269.0)	198.0 (151.0–240.2)	<0.001
**Platelet (10^9^/L), median (IQR)**	156.0 (116.0–202.0)	161.0 (121.0–204.0)	169.5 (120.8–219.0)	0.002
**PT (s), median (IQR)**	11.9 (11.3–12.6)	11.9 (11.4–12.6)	13.5 (13.0–14.2)	<0.001
**TT (s), median (IQR)**	19.3 (18.3–20.3)	19.3 (18.3–20.3)	17.6 (16.9–18.3)	<0.001
**Fibrinogen (mg/dl), median (IQR)**	2.4 (2.0–3.0)	2.4 (2.0–3.0)	2.8 (2.4–3.4)	<0.001
**APTT (s), median (IQR)**	27.4 (25.3–29.9)	27.3 (25.4–29.8)	37.2 (34.7–40.0)	<0.001
**Tumor number**				<0.001
**1**	4749 (80.1%)	1198 (80.8%)	437 (86.0%)	
**2**	729 (12.3%)	181 (12.2%)	54 (10.6%)	
**3**	170 (2.9%)	36 (2.4%)	0 (0.0%)	
**4**	63 (1.1%)	17 (1.1%)	0 (0.0%)	
**5**	217 (3.7%)	51 (3.4%)	17 (3.3%)	
**Tumor diameter (cm), median (IQR)**	5.2 (3.4–8.5)	5.3 (3.5–8.3)	4.5 (3.0–7.5)	<0.001
**Tumor capsule, n (%)**	4309 (72.7%)	1070 (72.2%)	399 (78.5%)	0.003
**Tumor differentiation, Ⅰ/Ⅱ, n (%)**	4845 (81.7%)	1230 (82.9%)	207 (40.7%)	<0.001
**Tumor thrombus, n (%)**	802 (13.5%)	181 (12.2%)	95 (18.7%)	<0.001
**Satellite nodules, n (%)**	2594 (43.8%)	650 (43.8%)	139 (27.4%)	<0.001
**MaVI, n (%)**	1004 (16.9%)	228 (15.4%)	142 (28.0%)	<0.001
**MVI, n (%)**	2279 (38.4%)	562 (37.9%)	314 (61.8%)	<0.001
**Major resection, n (%)**	4728 (79.8%)	1169 (78.8%)	425 (83.7%)	0.026
**Blood transfusion, n (%)**	645 (10.9%)	158 (10.7%)	70 (13.8%)	0.040

IQR, interquartile range (25%–75%).

EHSH, Eastern Hepatobiliary Surgery Hospital; MHH, Mengchao Hepatobiliary Hospital; FLD, fatty liver disease; HBV, hepatitis B virus; AFP, alpha-fetoprotein; TBIL, total bilirubin; PT, prothrombin time; HBG, hemoglobin concentration; GGT, gamma-glutamyl transpeptidase; TT, thrombin time; APTT, activated partial thromboplastin time; MaVI, macrovascular invasion; MVI, microvascular invasion; ALBI grade, albumin-bilirubin grade.

*Comparison between EHSH and MHH cohorts.

### Predictive Performance

**Figure 1:**
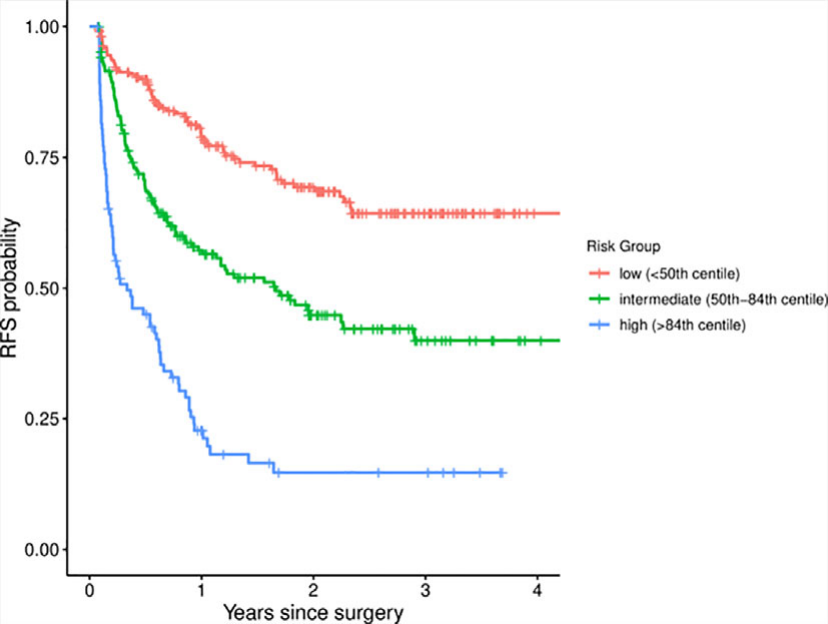
Kaplan-Meier curves for different risk groups among MHH patients. MHH, Mengchao Hepatobiliary Hospital.

The discriminatory performance of the four models was assessed with the Harrell’s c-index (**Table 2**). The c-index of the Cox regression model in three cohorts were 0.704 (EHSH derivation), 0.700 (EHSH validation), and 0.703 (MHH validation). Among four models, XGBoost achieved the highest c-index in the internal validation cohort (c-index: 0.713, P < 0.05, all comparisons). The c-index of XGBoost in the external validation cohort of MHH is 0.697, no statistically significant difference from those of CPH, DeepSurv, and RSF (0.703, P = 0.470; 0.700, P = 0.616; and 0.699, P = 0.672; respectively). Meanwhile, XGBoost model outperformed the Early Recurrence After Surgery for Liver tumor (ERASL) model (c-index: 0.672, P < 0.001; 0.673, P < 0.001; and 0.679, P = 0.185) in all three cohorts with our dataset. Thus, XGBoost was employed for the following demonstration and analysis. KM curves of the external validation dataset (**Figure 1**) indicated good discriminative ability of XGBoost to categorize patients into three risk groups after resection: low risk, intermediate risk (p < 0.001 in comparison to the low-risk group), high risk (p < 0.001 in comparison to the intermediate-risk group).

**Table 2:** Predictive performance (c-index with 95% CI) of the different models.

	EHSH derivation	EHSH validation	MHH validation
CPH	0.704 (0.694–0.712)	0.700 (0.683–0.719)	0.703 (0.671–0.733)
DeepSurv	0.697 (0.687–0.707)	0.698 (0.682–0.718)	0.700 (0.663–0.737)
RSF	0.702 (0.691–0.713)	0.704 (0.685–0.722)	0.699 (0.665–0.730)
XGBoost	0.704 (0.695–0.714)	0.713* (0.698–0.731)	0.697 (0.661–0.728)
ERASL	0.672 (0.663–0.681)	0.673 (0.654–0.690)	0.679 (0.636–0.714)

EHSH, Eastern Hepatobiliary Surgery Hospital; MHH, Mengchao Hepatobiliary Hospital; CPH, Cox Proportional Hazards Regression; DeepSurv, Deep Learning-Based Survival Model; RSF, Random Survival Forest; XGBoost, Extreme Gradient Boosting; ERASL, Early Recurrence After Surgery for Liver tumor models

*p < 0.001 in comparison to DeepSurv and RSF models, p = 0.008 in comparison to CPH model.

**Figure 2:**
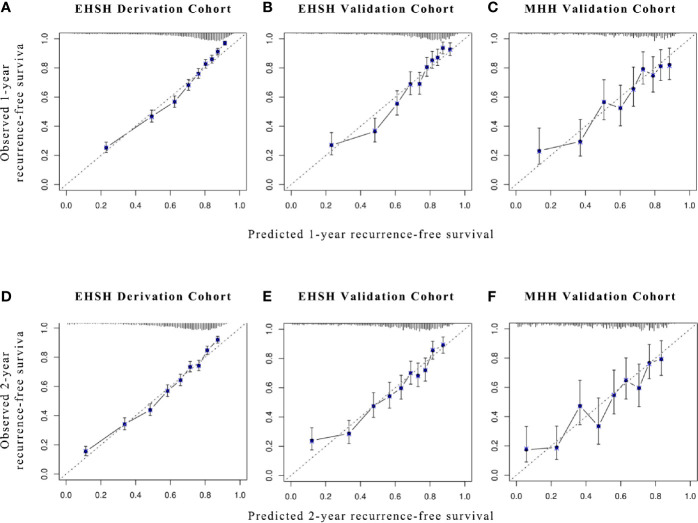
Calibration plots for XGBoost models in predicting 1- and 2-year RFS. Calibration plots for **(A, D)** EHSH derivation cohort, **(B, E)** EHSH validation cohort, and **(C, F)** MHH validation cohort in predicting 1-year **(A–C)** and 2-year RFS **(D–F)**. RFS, recurrence-free survival.

As shown in **Figure 2**, the calibration plots demonstrated a satisfying agreement between predictions made by XGBoost and actual patient outcomes in all datasets.

### Models and Feature Importance in Different Time Intervals

We established the XBGoost model in different time intervals, including 0 to 1 year, 1 to 2 year, 2 to 3 year, and 3 to 5 years, to examine the dynamics of feature importance in HCC patients. The specific predictive performance measurements using c-index and 95% CI for each time slot are listed in **Table 3**.

**Table 3:** Predictive performance (c-index with 95% CI) of XGBoost in four time intervals.

Time Intervals	EHSH derivation	EHSH validation	MHH validation
0–1 year	0.736 (0.726–0.748)	0.751 (0.731–0.772)	0.712 (0.671–0.751)
1–2 years	0.608 (0.579–0.632)	0.551 (0.498–0.604)	0.667 (0.553–0.757)
2–3 years	0.581 (0.545–0.622)	0.571 (0.508–0.641)	NA
3–5 years	0.565 (0.530–0.605)	0.689 (0.625–0.751)	NA

EHSH, Eastern Hepatobiliary Surgery Hospital; MHH, Mengchao Hepatobiliary Hospital; NA, not available.

The variables with the top 10 importance scores are shown in **Table 4**. During 0 to 1 year after resection, the importance score of tumor thrombus (defined as the tumor extending into a vessel, typically portal vein) was 103.01, substantially higher than scores of other factors, such as tumor diameter (33.94), gamma-glutamyl transpeptidase (GGT) (20.25), and tumor capsule (19.22). For 1 to 2 year, tumor number (13.39) was the most important variable related with patient outcomes, followed by resection type (major resection 13.22), tumor thrombus (13.04), and tumor diameter (12.36). In the latter two intervals, apart from tumor number, HBV infection was found to be a relatively important variable. HBV-DNA load has the third highest importance score for 2 to 3 years and HBsAg ranked first in the last period. Furthermore, smoking, an unhealthy lifestyle, was also associated with late recurrence.

**Table 4:** Feature importance during the four time intervals.

No.	0–1 year		1–2 years		2–3 years		3–5 years	
	Features	Importance Score	Features	Importance Score	Features	Importance Score	Features	Importance Score
1	Tumor thrombus	103.01	Tumor number	13.39	Tumor number	8.00	HBsAg	13.26
2	MaVI	37.47	Major resection	13.22	Smoking	7.99	Prealbumin	11.28
3	Tumor diameter	33.94	Tumor thrombus	13.04	HBV-DNA load	7.48	Smoking	8.94
4	MVI	33.63	Tumor diameter	12.36	HBeAg	7.20	Tumor number	8.67
5	GGT	20.25	Satellite nodules	12.01	Major resection	7.14	Age	8.41
6	AFP	19.55	HBV-DNA load	11.89	MaVI	6.95	Platelet	8.40
7	Tumor capsule	19.22	GGT	11.89	Alcohol consumption	6.70	AFP	8.26
8	Blood transfusion	18.21	Albumin	9.94	MVI	6.68	PT	8.21
9	Major resection	17.57	Tumor capsule	9.58	Platelet	6.58	Tumor diameter	8.17
10	Tumor number	15.10	Platelet	8.98	Tumor diameter	6.52	MaVI	8.13

AFP, serum alpha-fetoprotein; GGT, gamma-glutamyl transpeptidase; HBV, hepatitis B Virus; MaVI, macrovascular invasion; MVI, microvascular invasion; PT, prothrombin time.

### The Pattern of Recurrence Risk

**Figure 3:**
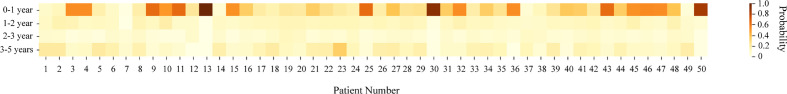
Risk heat map for 50 randomly selected patients.

Using the XGBoost model in different time intervals, a risk heat map covering four time intervals was developed that visually depicts a patient’s risk of tumor recurrence, metastasis or death after undergoing curative liver resection. In general, individual heat map indicated a trend of relatively high recurrence risk in 0 to 1 year and 3 to 5 years after surgical resection (**Figure 3**).

## Discussion

HCC is one of the most common malignancies worldwide. Though curative resection offers the best prognosis for patients, disease recurrence remains a major obstacle to the long-term survival of patients (21). Moreover, little is known about the potential risk and peak time periods of HCC recurrence after curative surgery (22, 23). We therefore conducted this research to mediate this gap. In this study, the risk prediction model based on the XGBoost algorithm showed the best c-index in the EHSH validation set. To observe the recurrence risk of individual patients at different time intervals post-surgery, a heat map was constructed based on the XGBoost model for 50 randomly selected HCC patients. The majority of patients had a similar trend of postoperative recurrence that risks in 0 to 1 and 3 to 5 years after surgery were higher than those in 1 to 2 and 2 to 3 years.

In the past few years, several scoring systems have been developed for estimating HCC recurrence risk and stratifying patients. These systems have primarily selected significant clinical parameters through multivariate analyses and constructed conventional Cox proportional hazard models based on the limited risk factors (24–26). One of the important assumptions for Cox proportional hazards regression is that each variable makes linear contribution to model. However, in clinical studies, multiple risk factors usually have non-linear effects with recurrence-free survival, especially in cancer studies (16, 27, 28). Due to this reason, the previous models might fail to show goodness-of-fit and to make accurate prediction. Machine learning algorithms are probably superior than conventional CPH because they can fit more sophisticated non-linear relationship. According to our attempts of building different models, the XGBoost model did better prediction of liver recurrence.

Apart from an individualized heatmap for illustrating recurrence risk, a feature importance analysis was conducted based on the XGBoost model and was used to evaluate dynamics of variables contributing to the interesting outcome. Specifically, tumor characteristics, such as tumor thrombus, tumor number, tumor size, and tumor differentiation, contributed more to the model’s predictive performance in our study. In addition, macrovascular invasion (MaVI), microvascular invasion (MVI), gamma-glutamyl transpeptidase (GGT), intraoperative blood transfusion and major resection also showed a more significant contribution to the predictive performance of the model. Furthermore, smoking as an unhealthy lifestyle also hampered prognosis of HCC patients. These findings are supported by previous research as follows.

Firstly, previous studies found that patients with portal vein tumor thrombosis (PVTT) usually decreased liver function reserves, which was a high-risk factor for disease progression and recurrence (29, 30). In addition to tumor thrombus, tumor volume is also associated with HCC recurrence. In another study, tumor volume was shown to be a predictor of HCC recurrence after liver transplantation (31). A clinical study in Korea confirmed that the maximal size of HCC and the number of tumors were significantly correlated with the recurrence of HCC after liver transplantation (32). In line with our results, MVI was also a unique parameter assessed in the ERASL, SLICER, SS-CLIP, and Korean models (5–8). The dissemination and spread of tumors through micro-vessels may explain the advanced tumor stage, tumor progression, and worse outcomes (33–35).

Secondly, perioperative blood transfusions were independently associated with survival and cancer recurrence after surgical resection (36). A meta-analysis found that allogeneic blood transfusions were associated with poor clinical prognoses in patients with HCC who underwent radical hepatectomy (37). The association between major resection and blood loss as well as RFS of HCC patients has been examined: the more complicated hepatectomy is, the more likely patients are to suffer from intraoperative blood loss, leading to shorter time to recurrence (38).

Thirdly, liver function presented by GGT was another crucial prognostic factor to predict tumor recurrence (39). GGT was first found to modulate the metabolism of glutathione (GSH) and facilitate amino-acid recovery for GSH synthesis (40). Recently, GGT was reported to be involved in tumor initiation, progression, and invasion. As such, GGT may induce the production of endogenous reactive oxygen species (ROS), leaving cells exposed to persistent oxidative stress, leading to DNA damage and tumor growth (41, 42).

Moreover, smoking was associated with an increased risk of HCC (43, 44) and disease-free survival of patients who underwent resection (45). In the current study, we found that smoking was associated with a recurrence risk of 2 to 3 and 3 to 5 years after HCC. The underlying mechanism might be that nicotine increases the expression of α-7-nicotinic acetylcholine receptor (α-7-nAChR), leading to recurrence through the JAK2/STAT3 signaling pathway (46). A previous study found that the history and amount of smoking were both risk factors for the progressive recurrence of HBV-related HCC (47).

Finally, early disease recurrence (0–1 year) is often thought to be a result of intrahepatic metastases, while late recurrence is more likely to result from newly-onset tumors with multicenter origins (48, 49). In accordance with this theory, HBV-DNA load and HBsAg contribute significantly to HCC recurrence from two to five years in our study, which likely induce genomic alternations and pro-oncotic signaling for *de novo* HCC in the long term (50).

Our results suggest that clinicians can provide personalized management of recurrence risk after surgical resection in HCC patients based on information provided by heat maps and feature importance, which may improve postoperative survival outcomes. The risk heat map allows clinical teams to detect patients most at risk of HCC recurrence, schedule appointments for them in the “heat zones” that most likely for recurrence, and take interventions as needed. For example, clinicians may give greater attention to malignant characteristics of tumors, including the presence of tumor thrombus, larger tumor sizes, multiple tumor nodules, and micro- or macro-vascular invasion, if the heat map indicates a high risk within one year after surgery.

There are certain underlying limitations to our study. Firstly, our model is primarily based on two Chinese institutions of patients with HCC in hepatitis B virus-endemic areas. It is necessary to validate our model in international cohorts to extend our results to patients with HCC of various etiologies. Second, some other variables that may be associated with the prognosis of HCC patients, such as postoperative adjunctive therapies and serum inflammatory markers, were not evaluated in this study. In addition, further prospective studies with longer follow-ups are essential to extend the performance of our model further.

In summary, we have developed a model based on a machine learning algorithm that better predicts the risk of disease recurrence in individual patients following hepatic resection in a large population. We further applied this model to four time periods to describe patterns of HCC relapse, and to explore important risk factors. The heat map offers clinicians a decision support tool to identify individuals prone to recurrence, while also allowing clinicians to identify the prognostic factors, which are clinically useful in terms of individualized patient monitoring, surveillance, and management. Future prospective studies are needed to verify our conclusions.

## Data Availability Statement

The raw data supporting the conclusions of this article will be made available by the authors, upon reasonable request.

## Author Contributions

YH and HC contributed equally to the manuscript as they both took charge of study design and implementation, as well as drafting manuscript. YZ also participated in study design and literature review. Both ZL and HM conducted statistical and machine learning analysis. Corresponding author, JL, made a huge contribution to the manuscript in terms of revising the draft and reviewing the final version. All authors contributed to the article and approved the submitted version.

## Funding

This study was funded by Startup Fund for scientific research, Fujian Medical University (Grant Number: 2019QH1297).

## Conflict of Interest

The authors declare that the research was conducted in the absence of any commercial or financial relationships that could be construed as a potential conflict of interest.

## Supplementary Material

Supplementary Figure 1 Flowchart of inclusion and exclusion criteria and analysis strategy.

## Abbreviations

α-7-nAChR, 7-nicotinic acetylcholine receptor; APTT, Activated partial thromboplastin time; ALBI, Albumin-bilirubin grade; AJCC, American Joint Committee on Cancer; CPH, Cox Proportional Hazards Model; DeepSurv, Deep Learning-based Survival Model; ERASL, Early Recurrence After Surgery for Liver tumor model; EHSH, Eastern Hepatobiliary Surgery Hospital, Second Military Medical University; XGBoost, Extreme Gradient Boosting; FLD, Fatty liver disease; GSH, glutathione; HBG, Hemoglobin concentration; HCC, Hepatocellular carcinoma; HBV, Hepatitis B virus; MaVI, Macrovascular invasion; MHH, Mengchao Hepatobiliary Hospital, Fujian Medical University; MVI, Microvascular invasion; PVTT, Portal vein tumor thrombosis; PT, Prothrombin time; RSF, Random survival forest; ROS, Reactive oxygen species; RFS, Recurrence-free survival; AFP, Serum alpha-fetoprotein; SLICER, Singapore Liver Cancer Recurrence score; SS-CLIP, Surgery-Specific Cancer of the Liver Italian Program; TT, Thrombin time; TBIL, Total bilirubin.
