# Supplementary figures and images for: Development and Validation of a Machine Learning Prognostic Model for Hepatocellular Carcinoma Recurrence After Surgical Resection
Source: Front Oncol. 2021 Feb 1;10:593741. doi: 10.3389/fonc.2020.593741 (PMC7882739; doi:10.3389/fonc.2020.593741)

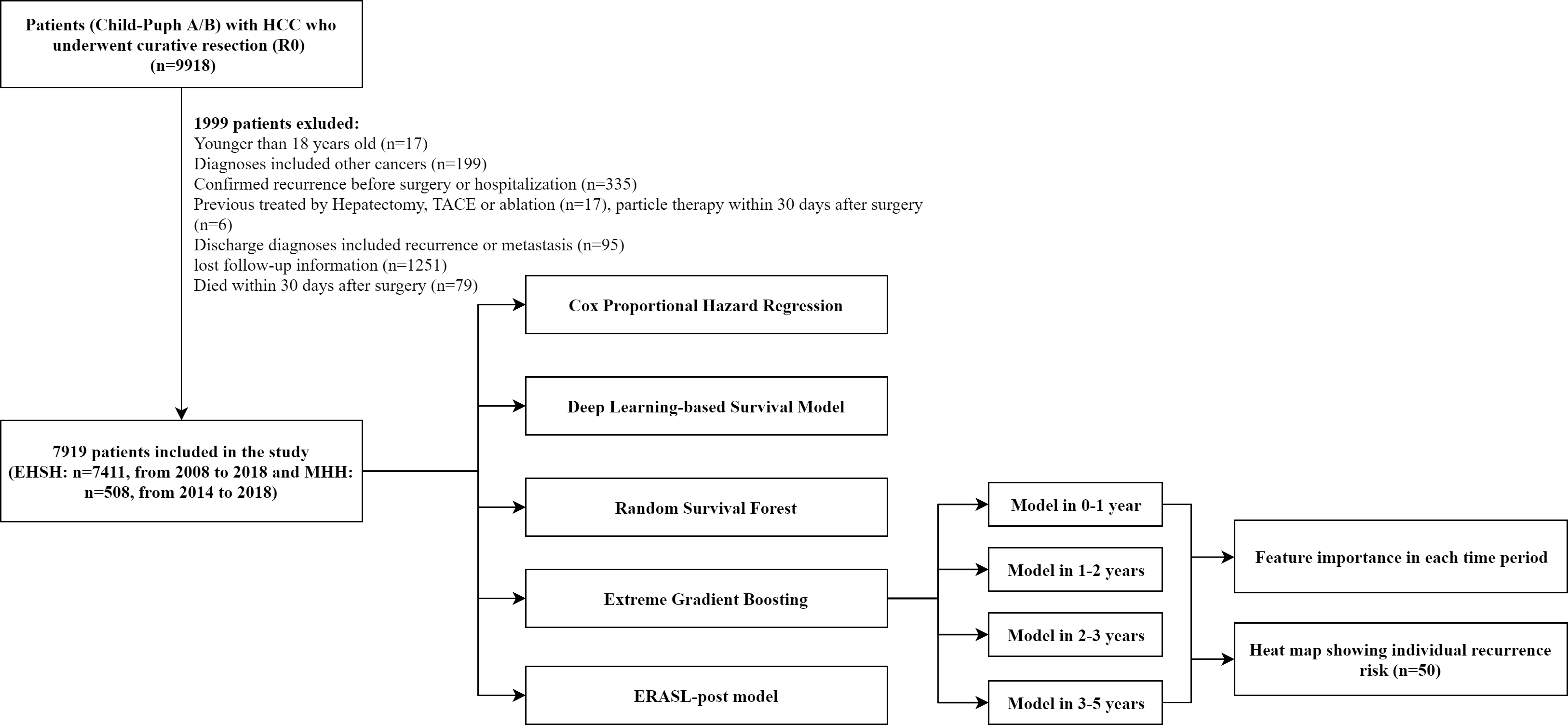

Supplement: Supplementary Figure 1 — Flowchart of inclusion and exclusion criteria and analysis strategy. [file Image_1.png]
