# Supplementary material for: Development and Validation of a Machine Learning Prognostic Model for Hepatocellular Carcinoma Recurrence After Surgical Resection
Source: Front Oncol. 2021 Feb 1;10:593741. doi: 10.3389/fonc.2020.593741 (PMC7882739; doi:10.3389/fonc.2020.593741)
Supplement: Supplementary file 2 [file Table_1.docx]

**Supplementary Table 1.** Recurrence-free survival (RFS) rates (with 95% CI) of the three cohorts

| **Cohort** | **1 Year** | **2 Year** | **3 Year** | **5 Year** |
| --- | --- | --- | --- | --- |
| **EHSH Derivation** | 70.2% | 60.3% | 54.5% | 45.2% |
|  | 69.1-71.5% | 59.0-61.7% | 53.1-55.9% | 43.6-46.8% |
| **EHSH Validation** | 69.8% | 60.0% | 54.1% | 45.3% |
|  | 67.4-72.2% | 57.4-62.7% | 51.4-57.0% | 42.2-48.6% |
| **MHH Validation** | 60.4% | 50.2% | 46.2% | NA |
|  | 56.1-65.1% | 45.5-55.3% | 41.3-51.8% | NA |

EHSH: Eastern Hepatobiliary Surgery Hospital; MHH: Mengchao Hepatobiliary Hospital; NA: Not available
